# The progressive trend of modeling and drug screening systems of breast cancer bone metastasis

**DOI:** 10.1186/s13036-024-00408-5

**Published:** 2024-02-05

**Authors:** Hanieh Kolahi Azar, Maliheh Gharibshahian, Mohammadreza Rostami, Vahid Mansouri, Leila Sabouri, Nima Beheshtizadeh, Nima Rezaei

**Affiliations:** 1 Department of Pathology, Tabriz University of Medical Sciences, Tabriz, Iran; 2 Department of Tissue Engineering, School of Medicine, Shahroud University of Medical Sciences, Shahroud, Iran; 3 Division of Food Safety and Hygiene, Department of Environmental Health Engineering, School of Public Health, Tehran University of Medical Sciences, Tehran, Iran; 4 Food Science and Nutrition Group (FSAN), Universal Scientific Education and Research Network (USERN), Tehran, Iran; 5 Gene Therapy Research Center, Digestive Diseases Research Institute, Shariati Hospital, Tehran University of Medical Sciences, Tehran, Iran; 6 Department of Tissue Engineering and Applied Cell Sciences, School of Paramedicine, Guilan University of Medical Sciences, Rasht, Iran; 7 Department of Tissue Engineering, Faculty of Advanced Medical Sciences, Tabriz University of Medical Sciences, Tabriz, Iran; 8 Regenerative Medicine Group (REMED), Universal Scientific Education and Research Network (USERN), Tehran, Iran; 9 Department of Immunology, School of Medicine, Tehran University of Medical Sciences, Tehran, Iran; 10 Research Center for Immunodeficiencies, Children’s Medical Center, Tehran University of Medical Sciences, Tehran, Iran; 11 Network of Immunity in Infection, Malignancy and Autoimmunity (NIIMA), Universal Scientific Education and Research Network (USERN), Tehran, Iran

**Keywords:** Breast cancer, Bone metastasis, Tumor spheroids, Cancer modelling, Regenerative medicine

## Abstract

Bone metastasis is considered as a considerable challenge for breast cancer patients. Various in vitro and in vivo models have been developed to examine this occurrence. In vitro models are employed to simulate the intricate tumor microenvironment, investigate the interplay between cells and their adjacent microenvironment, and evaluate the effectiveness of therapeutic interventions for tumors. The endeavor to replicate the latency period of bone metastasis in animal models has presented a challenge, primarily due to the necessity of primary tumor removal and the presence of multiple potential metastatic sites.

The utilization of novel bone metastasis models, including three-dimensional (3D) models, has been proposed as a promising approach to overcome the constraints associated with conventional 2D and animal models. However, existing 3D models are limited by various factors, such as irregular cellular proliferation, autofluorescence, and changes in genetic and epigenetic expression. The imperative for the advancement of future applications of 3D models lies in their standardization and automation. The utilization of artificial intelligence exhibits the capability to predict cellular behavior through the examination of substrate materials' chemical composition, geometry, and mechanical performance. The implementation of these algorithms possesses the capability to predict the progression and proliferation of cancer. This paper reviewed the mechanisms of bone metastasis following primary breast cancer. Current models of breast cancer bone metastasis, along with their challenges, as well as the future perspectives of using these models for translational drug development, were discussed.

## Introduction

Breast cancer is the most common cancer worldwide, ranking first in most diagnosed malignancies in women and having the highest malignancy-related mortality [1, 2]. Despite increasing attention to screening and early detection of breast cancer, plenty of patients were only diagnosed at late stages, when multiple metastases could have been made [3, 4]. The most common sites of breast cancer metastasis include bones, lungs, liver, and brain, the most common of which is the bones and skeletal system, which account for about 65–80% of metastasis in patients with metastatic breast cancer [5, 6]. Approximately 70% of patients with advanced breast cancer developed bone metastases at the time of diagnosis [7]. Metastasis, defined by the spread and growth of malignant cells to distant organs, yields a high mortality and morbidity burden [8, 9]. Generally, when breast cancer is accompanied by metastasis, the patient’s prognosis dramatically decreases [10, 11]. Patients with metastatic breast cancer had a median overall survival of 2–3 years, with a 27% overall 5-year relative survival rate [12].

Currently, there are several treatments for bone metastases, including bone-modifying agents; however, their efficacy and side effects remain a matter of controversy [13–16]. One of the reasons for this debate could be the lack of a comprehensive understanding of the complex interplay between various involved compartments (i.e., circulating malignant cells, tumor, and bone microenvironment) [17, 18]. Therefore, understanding the mechanism through which the various stages of bone metastasis occur could play a pivotal role in the development of effective and promising treatments for advanced breast cancer.

Bone metastases are classified into two categories: osteolytic and osteoblastic, based on the interaction of malignant cells, osteoblasts, and osteoclasts. Typically, breast cancers result in osteolytic lesions; however, subsequent activation of osteoblasts could cause mixed lesions, including both the destruction of affected bone and the construction of new bone [19, 20]. Although the metastatic pattern of certain tumors depends on the characteristics of the primary cancer, there is increasing evidence of the role of the metastasis-target microenvironment in the pathophysiology of metastatic breast cancer [21]. Migrated malignant mammary cells induce the upregulation of growth factors and other cytokines in the bone microenvironment, leading to the activation of osteoclasts and inhibiting the differentiation of osteoblasts. Subsequently, pathologic bone resorption and the secretion of morphogens are responsible for the progression of the imbalance between bone formation and resorption. Moreover, chemoattractants secreted by osteocytes further direct the circulating malignant cells towards the lesions [22, 23].

As mentioned above, the development of breast cancer bone metastasis would depend on a complex interaction of various factors, which, through spatial cross-talk, eventually led to the formation of metastatic lesions in the human body [24]. Although the lack of a valid and inclusive model that could comprehensively and authentically mimic the behavior of the effector factors outside of the human body hampers the development and assessment of bone-targeted therapeutics and reduces the translational efficacy of laboratory-effective drugs [24]. Thus, in this paper, we review the mechanisms of bone metastasis following primary breast cancer. Next, we reviewed current models of breast cancer bone metastasis along with their challenges. Finally, we demonstrated the future perspectives of using these models for translational drug development.

## Bone metastatic microenvironments

### Bone extracellular matrix

The extracellular matrix (ECM) is a key mediator of cancer incidence, progression, and metastasis [25]. The bone extracellular matrix is a dynamic milieu composed of organic and inorganic materials. The bone ECM acts in multiple inherent (e.g., cell adhesion) or distal (e.g., calcium homeostasis) biological processes [26, 27]. The extremely high strength of bone comes from the nanocomposite structure of the bone [28]. Collagen, elastin, and polysaccharides are the major organic materials within the bone structure. Collagenous contents of various structures and molecular weights play a key role in hydroxyapatite nucleation and growth [29].

Non-collagenous proteins (about hundreds of proteins) reside in the bone ECM. They can be produced by the cells or captured from the medium by electrostatic interactions. Matrix metalloproteinases (MMP), sialoprotein, fibronectin, proteoglycans, and osteocalcin are the most important members that directly govern cellular behaviors. MMP directs matrix remodeling and impulses mesenchymal stem cells (MSCs) to osteoblasts and osteoblasts to osteocytes [30, 31]. Sialoprotein, a glycosylated and sulfated protein, interacts with cells and hydroxyapatite through RGD [32]. Proteoglycans are made from a core protein and glycosaminoglycan (GAG) family chains, which have a major role in osteogenesis. In detail, proteoglycans serve as a setting to host and release growth factors and cytokines owing to their charged characteristics [33]. Breast cancer cells expressing MMP [34], sialoproteins [35–37], osteocalcin [36] represent metastatic activity.

Inorganic phases of the bone possess a platelet-like morphology dispersed within the organic matrix. The mineral portion of the bone resembles the hydroxyapatite structure (Ca_10_(PO_4_)_6_(OH)_2_); however, the Ca/P ratio varies due to multiple anion (e.g., Cl^−^) and cation (e.g., Mg^2+^) inclusions. Of note, the ionic substitution endows spectacular structure with individual biodegradation and bioactivity [38]. Namely, fluoride and carbonate enhance and decrease the crystallinity of bone, respectively [38]. Indeed, the constitution of the bone changes as a function of age, sex, disease, and nutrition [39].

Osteoblasts (4–6%), osteoclasts, osteocytes (90–95%) and bone lining cells are the major cells residing within the bone ECM. Mesenchymal stem cells convert into osteoclasts, osteoblasts, and bone-lining cells upon receiving a biochemical or biomechanical stimulus. In general, osteoblast proliferation/differentiation and osteoclast apoptosis/differentiation determine the bone formation ratio. Transforming growth factor-β (TGF-β), insulin-like growth factors (IGF), and fibroblast growth factors (FGF) are the most common growth factors modulating osteoblast proliferation. Meanwhile, apoptosis of the osteoclasts is mostly driven by TGF-β and drugs such as bisphosphonates and estrogen. Osteoblasts interact with ECM through integrin families such as α_v_β_3_ (binds to RGD) / α_2_β_1_ (binds to collagen) [40, 41]. Meanwhile, osteocytes employ β_1_ and β_3_ integrins for adhering to the ECM [42, 43].

## Mechanism of bone metastasis

Approximately two-thirds of patients with breast cancer are diagnosed with bone metastases. Skeletal-related events (SRE) are pathologically occurring fractures that demand surgery and cancer treatment simultaneously. Patients with > 2 cm breast cancer tumors that reside in the T and N stages were more prone to bone metastasis [44]. In a study on 295,213 patients, it was reported that hormone receptor (HR +)/human epidermal growth factor 2(HER2 +) had a high risk of bone metastasis [45]. In clinics, patients diagnosed with bone metastasis are commonly treated with bone-modifying agents (e.g., denosumab, zoledronic acid) to prevent skeletal-related events and hypercalcemia [45].

Breast cancer cells that reside in the bone ECM induce osteoclast formation by releasing tumor necrosis factor-α (TNF-α), parathyroid hormone-related peptide (PTH-rP), prostaglandin E2 (PGE2), interleukins, and leukemia inhibitory factor (LIF). PTH-rP induces osteoclast activation by receptor activator of nuclear factor-κB ligand (RANKL) cytokine secretion, which targets the RANK receptor on osteoclasts. The bone metastatic behavior of breast cancer cells was eliminated following PTH-rP neutralization [46]. Of note, RANKL is among the major inducers of osteoclast differentiation in monocytes [47] and myeloid cells. Meanwhile, RANKL expression in breast cancer cells aggravated their metastatic behavior and bone resorption via MMP1 and IL-11 activation [48, 49]. Also, cancer cells secrete TNF-α and interleukins (IL-1, IL-6, IL-8, and IL-1) which elicit osteoclast activation and migration through RANKL expression.

The bone resorption following the re-location of cancer cells brings TGF-β release, which manipulates the inhibition of osteoblast differentiation and aggravates the osteolytic activity of osteoclasts. TGF-β activates the osteolytic and metastasis-inducers PTH-rP and IL-11 [46]. PTH-rP secretion occurs following the p38 mitogen-activated protein (MAP) kinase pathway. Also, TGF- β regulates connective tissue growth factor (CTGF)/IL-11 [50], CXCR4/MMP-1 [51], and cyclooxygenase (COX)-2 [52]. Of note, TGF-β has the principal role in the epithelial-mesenchymal transition (EMT) of breast cancer cells [53].

The role of interleukins as pro-osteoclastogenesis agents is well defined by the regulation of two signaling pathways: RANK/RANKL/OPG [54] and JAK1/STAT3 signaling [55]. As lately discussed, elevated concentrations of TGF- β in bone destruction procedures increase IL production by bone marrow stromal cells, as evidenced by increased levels of IL-11 and IL-8 [56, 57]. Moreover, IL-6 serum levels vary as a function of bone metastasis and the number of additional metastasized organs [58]. The role of IL-1β [59, 60], IL-6, IL-8, and IL-11 [60] in encouraging metastatic behavior is evidenced.

## Advanced modeling and drug screening systems

Solid tumors in the human body have a unique structure and behavior that enable them to escape the immune system and sometimes align the immune system with themselves to be distributed throughout the body [61]. On the other hand, they are resistant to treatment and require extensive research to discover and introduce new drugs [62].

Human body conditions' difficulties during tumor development and its interaction in laboratory simulations and the lack of a distinct tumor microenvironment (TME) model lead to limited approved anti-cancer medicine on the market [63]. So far, extensive and comprehensive efforts have been made to model tumors for drug screening and conduct more studies on tumor structure and behavior [64, 65].

**Fig. 1:**
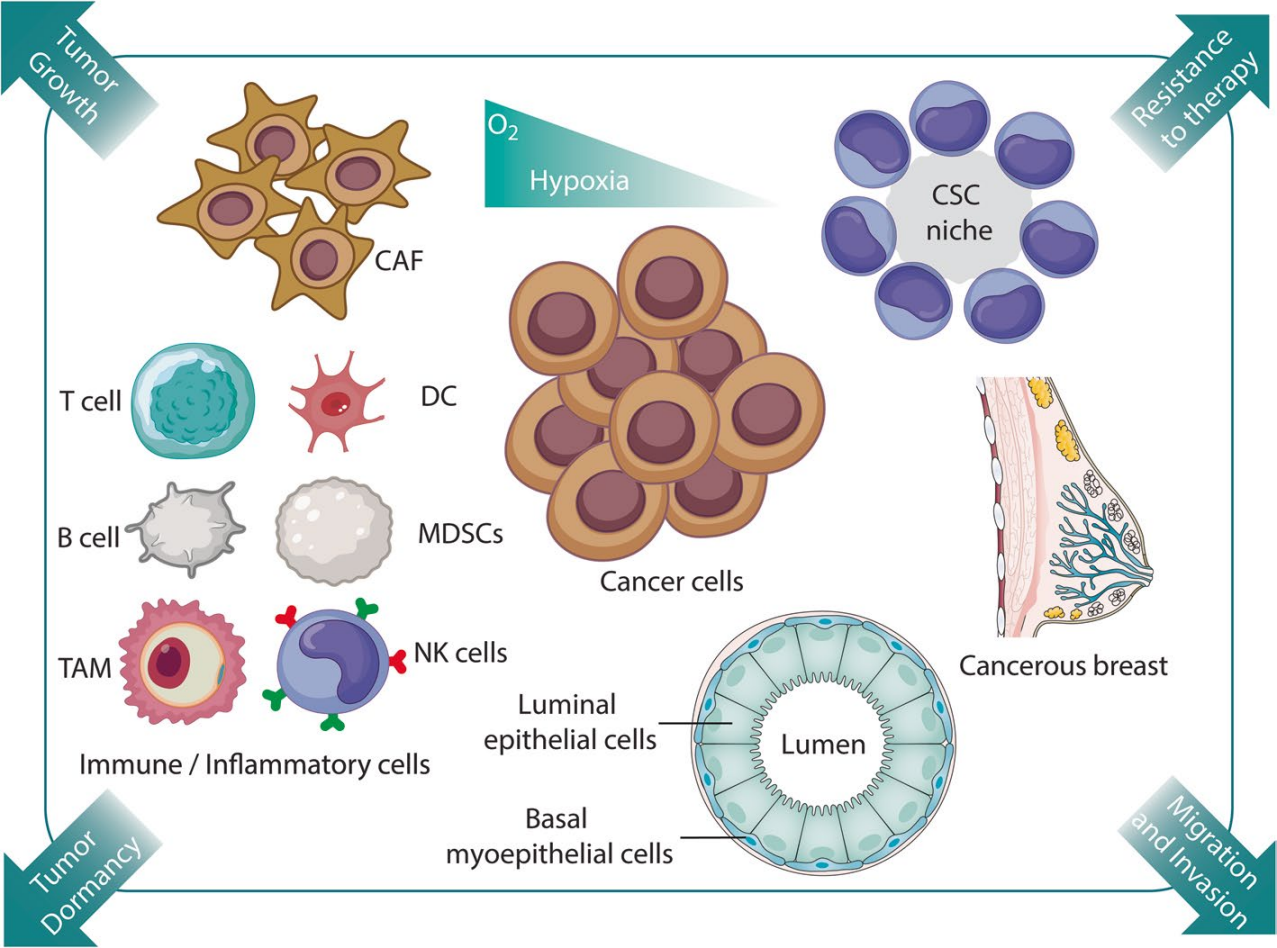
The tumor microenvironment consists of cancer stem cells (CSC), cancer-associated fibroblasts (CAFs), Dendritic cells (DC), myeloid-derived-suppressor cells (MDSCs), natural killer (NK), tumor-associated macrophages (TAM)

Various non-tumor cells, such as endothelial cells, fibroblast cells, and immune cells, are also present in the TME and interact with tumor cells (Fig. 1). As mentioned previously, endothelial cells are essential components involved in vascular formation and tumor metastasis [66]. Fibroblasts in TME have a high proliferation rate, ECM production, and secretion of carcinogenesis-enhancing cytokines [67]. Interestingly, immune cells stimulate and inhibit the proliferation, migration, and metastasis of cancer cells [68, 69]. After the tumor escapes from the immune system, TME affects the immune cells’ function and causes the immune cells to promote the tumor spread [70]. Tumor-associated macrophages increase the ability of cancer cells to destroy the endothelial barrier [71, 72].

A wide range of tumor models, from monolayer 2D to 3D models and animal models, have been proposed to study cancer biology, invasiveness, metastatic disease, and drug screening [73, 74]. Currently, 2D cultures on thermoplastics and animal models are routinely used for this purpose, although each has drawbacks that limit its usage. 2D monolayer culture is an inexpensive and uncomplicated method, but it cannot reconstruct the complex 3D structure and the tumor’s multicellular components, such as immune cells [75]. This model lacks cell–cell and cell-ECM interactions as well as the cell cycle and signaling necessary for cellular functions such as proliferation, differentiation, migration, drug metabolism, and drug sensitivity [76, 77]. Due to the cells' adequate access to oxygen and nutrients, the 2D model cannot mimic the tumor’s chemical and hypoxic gradients [75].

In a native tumor, the tumor structure (hypoxia, low nutrient intake, and low pH) causes overexpression of multi drug resistance (MDR) proteins by tumor cells, resulting in the resistance of the patient's tumor cells to a wide range of chemotherapy drugs [78]. In the 2D model, it is impossible to simulate the above conditions, and the drug reaches the tumor without any physical barriers. Therefore, this model’s predictive power is not so high, and its results are not reliable for transfer to the clinic [79, 80].

Animal models also face limitations, such as time-consuming, expensive, ethical concerns, and the need to use a minimum number of animals, along with the inability to mimic specific human biology and physiology [81]. The inability of animal models to mimic the biology and physiology of the human body has limited the translation of their results to humans. Only limited manipulations can be performed on animals, and many drug screening studies have failed at the clinical phase [82]. Therefore, it is necessary to utilize PDX and immunodeficient mouse models, which are very expensive [83]. Besides, the creation of animal models is a time-consuming process and not suitable for rapid assessments.

3D models as a bridge between 2D systems and animal models can overcome the above limitations. These 3D models have evolved and have been able to mimic several features of tumor tissue, such as morphology, the gradient of chemical and biological factors, the expression of pro-angiogenic proteins, MDR, and the interactions of cell–cell and cell-ECM, via different 3D tissue engineering methods [73, 74, 84, 85]. Depending on the materials and technology used to make 3D in vitro models of bone metastasis, there are several categories. Some of the significant 3D in vitro models of bone metastasis include spheroid culture systems, scaffold-based and hydrogel-based models, bioreactor-based models, microcarrier-based models, bioprinting, and metastasis-on-a-chip systems (Table 1).

**Table 1:** Various invitro models of bone metastasis of breast cancer

In vitro models of bone metastasis	Advantages	Disadvantage
Multicellular tumor spheroids	Simple, long-term culture, coculture, patient specific	Low throughput, high shear force, necrotic cells in the center of the cellular sphere, simple architecture
Organotypic multicellular spheroids	Preserve original tumor tissue's cellular interactions and body's physiological environment	More expensive, complicated, and time-consuming
Organoids	Biologically stable, high- throughput, simultaneously simulate the structure and function of healthy and tumor tissues, patient specific	Lacke vascularization and immune system complexity
3D hydrogels or scaffolds models	Simulate TME mechanical, structural, chemical, and physical signals, cells can migrate in three dimensions and interact with other cells, high-throughput screening, reasonable cost	Lack of accurate position of cells, vascular structures, and un-uniform distribution of cells
Bioreactor-based models	Adjustable and controllable, stimuli mechanical signals, cell–cell, and cell-ECM interactions	High space and cost for dynamic cell culture
Microcarrier-based models	Enhance cellular activity, improves drug resistance, Stimulates cell–cell and cell-ECM interactions	Simple structure, low vascular potential
Bioprinting	Automatic and accurate control of cell distribution, large-scale structures, high-efficiency, reproducibility, integration of permeable vascular networks, complex architecture, Custom made architecture, co culture	Expensive, difficult to be adopted to high-throughput screening
Metastasis-on-a-chip systems	Invivo-like structure, chemical gradient, and precise spatio-temporal control of TME	Expensive, difficult to be adopted to high-throughput screening
2D models	Simple and low cost	Few cell–cell and cell-ECM interaction, do not reproduce cellular complexity

### In vitro models

#### Multicellular tumor spheroids

Among the scaffold-free 3D models, multicellular spheroids have become more popular so far, and there are several techniques for their construction and development [86]. These models can either be produced from a single cell-derived cancer cell line or followed by a combination of cells in specific processes, such as co-culture. The co-culture method increases the spheroids' architectural complexity and simulates the tumor structure within the body as closely as possible [63, 87].

Tumors within the body have unique characteristics, both genetically and in the cell phenotype. The mimicry of them is essential for the discovery and study of new drugs in the laboratory. Multicellular spheroids depend on cells’ location in different layers, creating a concentration gradient of nutrients, oxygen, and pH, which in turn affects cell proliferation, gene expression, and related protein translation in different layers (Fig. 1) [88]. As a result, it increases the resistance of spheroids to anti-cancer drugs. Moving from the surface to the center of this cellular sphere, the cells’ proliferative power decreases. There are also necrotic cells in the center of the cellular sphere [63, 88].

**Fig. 2:**
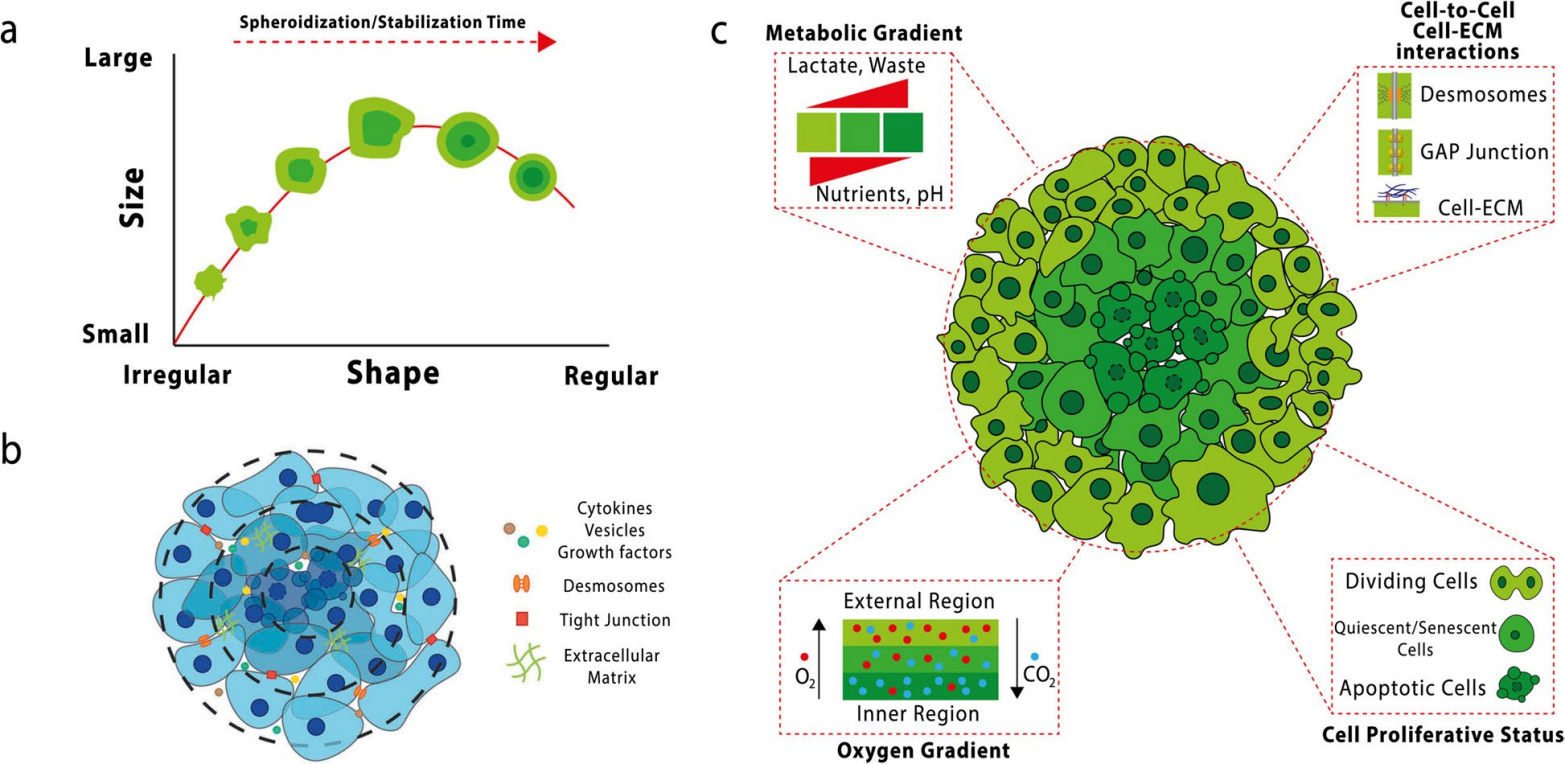
**a** Schematic representation of spheroid variation in shape and size over time, reprinted with permission from [88] (**b**) Schematic representation of a multicellular 3D spheroid cell culture, reprinted with permission from [63] (**c**) Main characteristics of the spheroid model, which is composed of several functionally differentiated areas and layers resulting from the impaired distribution of nutrients and oxygen. Tumor cells composing the spheroids interact with each other, developing a well-organized spatial architecture characterized by differences in phenotypic, functional, and metabolic status, reprinted with permission from [88]

As Fig. 2a illustrates, the shape and size of spheroids varied over time. Various parameters are affecting these indexes, while time is negligible. Multicellular 3D spheroid cell cultures, possessing cytokines, vesicles, and growth factors, can develop tight junctions and ECM (Fig. 2b). The distribution of oxygen in the inner cell layers is low, so such cells have different metabolisms and metabolites than others. Accordingly, they become resistant to drugs that are sensitive to pH and oxygen changes, and these drugs lose their effectiveness (Fig. 2c). Also, the inner cell layers are usually resistant to radiation therapy due to reduced reactive oxygen species. There is a higher expression of apoptotic-resistant genes in these cells [89].

Using a set of tumor cells along with stromal cells, such as fibroblasts, immune, and endothelial cells, as a collection of cells similar to the tumor microenvironment to model the spheroids creates a more heterogeneous and complex environment with cell–cell communication and signaling as well as cytokines and ECM secretions [63]. As a result, it increases tumor survival and promotes tumor cell metastasis [63].

#### Organotypic multicellular spheroids

In organotypic multicellular spheroids, the cells enter the spheroid condition directly based on the organ or tumor tissue culture. Thus, creating a comprehensive state of tumor modeling will preserve most of the original tumor tissue's cellular interactions. There are currently several techniques for organotypic culture; the most common is tissue slide culture [90].

Cell line culture merely for spheroid formation suffers from major obstacles, although it possesses significant advantages, such as high proliferation, inexpensiveness, and increased process speed [91]. Cell culture eventually affects gene expression and cell phenotype and causes them to deviate from their original states. Therefore, in this case, the resulting data can be somewhat unrealistic and deceptive.

This issue has been largely eliminated in organotypic culture, which provides closer information about the body's physiological environment [92]. However, organotypic culture methods are more expensive, complicated, and time-consuming than cell line culture methods. With this in mind, researchers are considering using and creating models that have both the benefits of cell and organ culture alone [63, 93]. Hence, they have started to produce new models called organoids [93].

#### Organoids

**Fig. 3:**
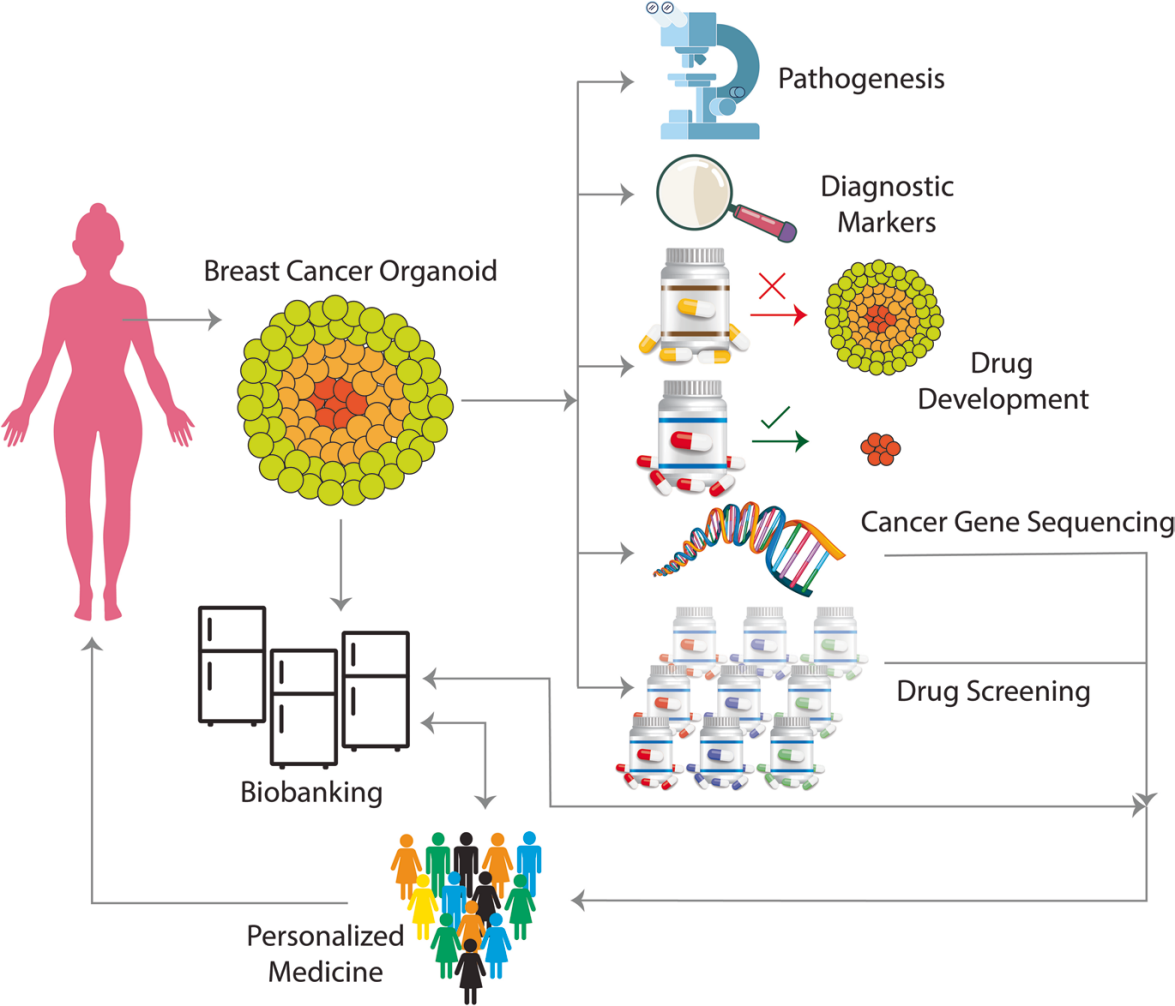
Organoids as tumor biobanks, can be prepared and stored from original tumor tissues. It is possible to obtain a biobank of various tumor subtypes from different individuals

Organoids are essential components in creating targeted organ 3D models. Studies show that stem cells have been used to design and fabricate organoids [88, 90]. Moreover, stem cells are more stable than other differentiated cells and cell lines under *ex-vivo* culture conditions and can form a heterogeneous set of stem, differentiated, and functional cells. Therefore, researchers can translate the data from them for drug screening at the clinic [63, 88, 90]. Using organoids, researchers can simultaneously simulate the structure and function of healthy and tumor tissues [94–96]. They create a miniature of the evolutionary process of cancer in the body. In this regard, organoids can be prepared and stored from the original tumor tissues. As a result, it is possible to obtain a biobank of various tumor subtypes from different individuals (Fig. 3).

However, the best-case study is preparing organoids from the patient's autologous tissue [97]. This method is being developed and studied under the title “personalized medicine”. Organoids are still in their infancy, and by knowing more about interstitial connections in their respective microenvironments, more structures, including blood vessels and types of inflammatory cells, can be added and engineered in these models [63, 98].

#### 3D hydrogel/scaffold models

3D matrices based on hydrogels or porous scaffolds are another approach to creating 3D tumor models. These structures encapsulate tumor cells in the matrix material. They are capable of providing TME mechanical, structural, chemical, and physical signals to cells. In hydrogels, tumor cells can move in all directions and interact with TME. The scaffold is more rigid than hydrogels, and cells can migrate in three dimensions and interact with other cells and TME components.

Polymeric scaffolds are first synthesized and then seeded with cells, while in hydrogels, the cells can mix with the polymers before the hydrogel formation [99]. Hydrogel-based 3D models are used for various purposes, such as the study of invasion [100], migration [101], angiogenesis [102], gene expression [103, 104], tumor growth [105], and drug screening [106]. They have a porous structure that allows the release of nutrients and metabolites in the model. Additionally, hydrogels have a reasonable cost, adjustability, and the ability to provide the mechanical and biochemical support necessary for cell survival and proliferation. They are adjustable for high-throughput screening (HTS) and, as will be discussed below, are also used in 3D printing techniques and microfluidics devices.

Synthetic hydrogels have sufficient flexibility in the design of tumor ECMs [107, 108]. Polyethylene glycol (PEG)-based hydrogels are studied for cell-instructive hydrogels [109]. These hydrogel systems’ physicochemical and biological properties could be designed at the molecular level [110]. The combination of bioactive molecules in the hydrogel increases cell adhesion and exposes them to degradation by proteases secreted by cells. Therefore, they can mimic ECM-cell interactions and tissue regeneration processes. These models present an acceptable ability to study morphogenesis and tumorigenesis [106, 109–111].

A scaffold is a biocompatible ECM to support the attachment, growth, and morphogenesis of cells. Porous scaffolds provide the ideal environment for reconstructing the native architecture and molecular crosstalk of tumor cells. Tumor cells cultured on scaffolds have a more aggressive phenotype and are more resistant to chemotherapy [112, 113]. However, scaffold-based cultures cannot accurately identify the position of cells in the structure. Lack of proper vascular structures in the tumor model for long-term perfusion in culture, limited throughput, limited HTS, the possibility of a lack of transparency, and un-uniform distribution of cells are other drawbacks of scaffold-based culture models [73, 114–116].

**Fig. 4:**
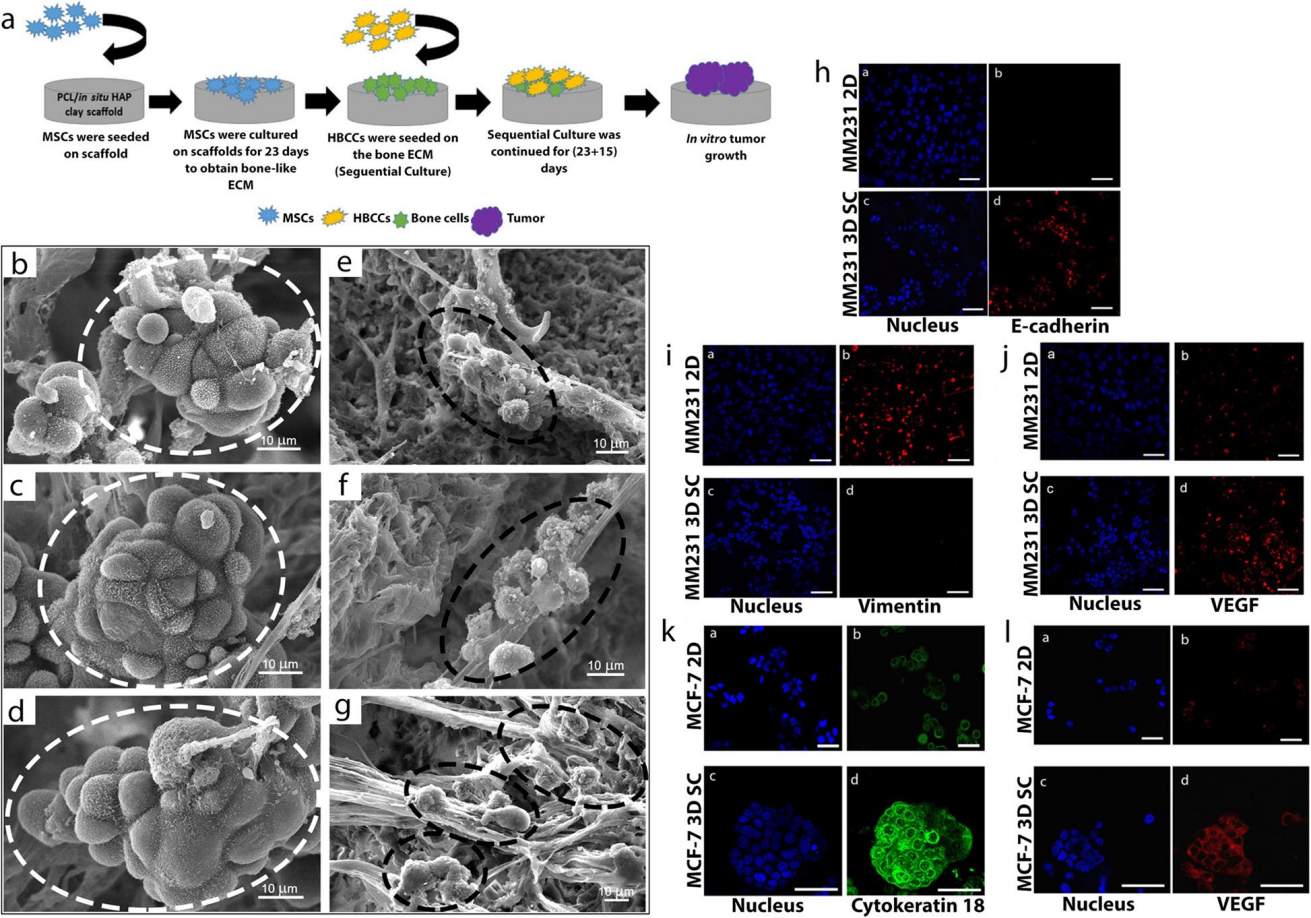
**a** Schematic showing the steps of sequential culture experiment; **b–d** Scanning electron microscope (SEM) micrographs of sequential culture of MCF-7 cells at days (23 + 5), (23 + 10), and (23 + 15; white circles/ellipses represent tumoroids); **e–g** Sequential culture of MM 231 cells at days (23 + 5), (23 + 10), and (23 + 15). (Black circles/ellipses represent disorganized clusters) (X + Y days: MSCs were cultured on polycaprolactone (PCL)/in situ HAPclay scaffolds for X days, then cancer cells were seeded and culture was continued for Y more days); **h–l** Representative immunofluorescence microscope images of MM 231 and MCF-7 cells cultured in 2D and 3D sequential culture after immunostaining for nuclei, **h** E-cadherin, **i** vimentin, **j, k** vascular endothelial growth factor (VEGF), and **l)** cytokeratin 18. Scale bar: 50 μm. Abbreviations: Human breast cancer cell (HBCC); human breast cancer cell (HBCC) lines MDA-MB-231 (MM 231). Reprinted with permission from [117]

Different natural and synthetic materials are used to make scaffold-based culture models. Kar et al. [117] used the freeze extraction method to construct scaffold-based models to evaluate breast cancer's bone metastasis. For this purpose, they used 3D scaffolds based on polycaprolactone and hydroxyapatite (HAP)/clay containing MSCs, human breast cancer cell (HBCC) lines MDA-MB-231 (MM 231), and MCF-7 cells (Fig. 4a). This 3D model provided a suitable microenvironment for cell–cell and cell–matrix interactions and maintained the metastasis potential of cells. The co-culture of MSCs and MCF-7 cells showed the formation of three-dimensional tumoroids and cancer metastasis (from mesenchymal to epithelial) (Fig. 4b-g). In addition, MDA-MB-231 cells with a high metastatic potential compared to MCF-7 cells with a low metastatic potential in these models had completely different behavior, migration potential, and invasion power, which indicates the ability of these models to evaluate the metastatic behavior of cancer cells (Fig. 4h-l).

Moreau et al. [118] used silk scaffolds containing morphogenetic protein-2 to model breast cancer bone metastasis. They seeded the scaffolds with BMSC cells for different periods and then implanted them orthotopically in NOD/SCID mice breast cancer models (containing the patient's femur). Then, SUM1315 cells were injected into the mammary glands of mice. SUM1315 cells were observed in all scaffold-bearing BMP-2 and scaffold-bearing MSCs. However, less migration was detected in the BMP-2-MSC scaffolds, which indicated the potential of the implanted scaffolds as a site of metastasis. Jin et al*.* [114] used acellular breast tissue as a scaffold for culturing tumor cells. The acellular breast tissue that has been attacked by breast cancer acts as a bioactive scaffold and supports epithelial-mesenchymal transmission [114]. In general, major drawbacks such as lack of perfusion, lack of shear stress, and the high cost of large-scale production have made the mere use of hydrogel- and scaffold-based systems inefficient.

#### Bioreactor-based models

The bioreactor-based models, in which the culture parameters are adjustable and controllable, are very similar to in vivo conditions. Closed bioreactor systems contain precision sensors connected to controller software that control dissolved oxygen levels, temperature, pH, nutrients, and metabolic input and output currents [119, 120]. Bioreactors can generate mechanical stimuli, appropriate signals, diffusion gradients, perfusion, cell–cell, and cell-ECM interactions [121]. Continuous perfusion prevents contamination and is a time-consuming process. Bioreactors are used to make various cell suspensions and play a significant role in feeding HTS by increasing microtumor production. In this regard, various bioreactors, including static systems, stirring, rotary, hollow-fiber, and perfusion, are used [120, 121].

Stirring and static bioreactors are the most common bioreactors in tumor engineering. Stirring bioreactors create a more realistic microenvironment for tumor cell growth in three dimensions. Perfusion bioreactors have great potential in surface studies and allow them to grow under dynamic conditions using fewer media [65]. This system is suitable for high-content imaging (HCI), but HTS is arduous and needs to transfer microtumors to multi-well plates. It requires a lot of space and cost for dynamic cell culture [120, 122].

#### Microcarrier-based models

Furthermore, microcarrier-based models can be used to co-culture multiple cell types in a spatial arrangement to grow cells that cannot aggregate and form a 3D structure spontaneously [65]. This approach is suitable for the reconstruction of solid tumors. The porosity of microcarriers provides more surface for cell growth. It is possible to develop microcarriers from both natural and synthetic polymers. Their diameter is between 50–400 μm, and due to their surface properties, density, and chemical composition, they can enhance adhesion, proliferation, and cellular activity [123]. This approach improves drug resistance and stimulates cell–cell and cell-ECM interactions.

Small microcarriers can be combined in microfluidic devices for precise and better control of experimental conditions. High-throughput microcarrier models support the cell phenotype, and cells can produce their matrix. These models are used to evaluate cellular functions, invasion, extravasation, gene expression, immune cell response, and drug screening [124–126]. However, they lack perfusion, shear stress, and vasculature [123, 127, 128].

The main challenge of the above 3D in vitro models is their overly simple structure and low vascular potential. Tumor spheroids and scaffold-based tumor models have size limitations due to the lack of vessels. Although they are reliable models for tumor genesis, they are not suitable for later tumor development stages. Most of these models lack the appropriate spatial distribution of tumor cells and ECM composition. Therefore, new methods, such as bioprinting and microfluidics, have been proposed for creating realistic tumor models to improve and accelerate the diagnosis and treatment of cancer.

#### Bioprinting models

Bioprinting is an emerging approach to 3D modeling of cancer that makes it possible to control the temporal and spatial distribution of cells and the spatial distance between cell types [129, 130]. 3D bioprinting is a sequential (layer-by-layer) method of distributing and depositing biological components, such as bioinks and cells, in a specific position to achieve computer-designed native tumor-like structures [131]. The ECM obtained by this method can be mixed with living cells before printing or loaded with cells after printing.

Bioprinting methods have unique capabilities, such as automatic and accurate control of cell distribution in three dimensions, creating large-scale structures, high-efficiency production of cancer models, reproducibility, integration of permeable vascular networks in engineered tissues, and accurate mimicry of the complex architecture and properties of TME [132]. Using 3D bioprinting methods, various properties of TME, such as stiffness and the spatial distribution of biochemical factors, can be adjusted. The effect of the TME on different stages of development, migration, metastasis, invasion, cell–cell interactions, and cell-ECM interactions of tumor cells can be investigated.

The bioprinting technique is useful in drug screening since it mimics the morphological and genetic profiles of tumors and the non-uniform deposition of various cell types and matrix components [133, 134]. Besides, this method makes it possible to create high cell densities and in vivo interactions. Bioink containing different cell types and ECMs can print materials into large-scale tumor tissues through high-density bioprinting. The bioink used to build tumor models must be carefully selected or designed. A suitable bioink must be printable, cross-linkable, and biocompatible [131]. The stability of the structure depends on its cross-linking. Table 2 demonstrates the printed models via the bioprinting technique and the biomaterials and cells used.

**Table 2:** Bioprinted breast cancer models and their used biomaterials and cells

Biomaterials	Cells	Significant inference	Ref
Methacrylated hyaluronic acid and methacrylated gelatin	Adipose derived mesenchymal stem/stromal cells and human epidermal receptor 2 positive breast primary breast cancer cells	Less sensitivity of 2D coculture of ADMSC and 21PT to doxorubicin, Low amount of cleaved caspase-3 positive cells in response to low-dose doxorubicin in ADMSC and 21PT-printed structures, When the thickness of the ADMSC layers was medium and thick, less cleaved Caspase-3 and less apoptosis were observed, increased DOX sensitivity of 21PT cells in bioprinted structures with LOX inhibitor Treatment	[135]
Polyethylene glycol with DMA on gelatin array Polyethylene	Michigan cancer foundation (MCF)-7 cells	Possibility of using cellular spheroids that mimic tumor structure for drug screening, bioprinted cellular spheroids have uniform cell seeding	[136]
Gelatin methacrylate and nanohydroxyapatite	Breast cancer cells and bone stromal cells	Possibility of studying the progression of breast cancer after metastasis by using the interaction of cancer cells with artificial bone microenvironment; Co-culture of breast cancer cells and bone stromal cells increases the growth of cancer cells, inhibits the growth of osteoblasts, increases vascular endothelial growth factor (VEGF) secretion, and decreases the alkaline phosphatase activity of osteoblasts	[137]
Hydroxyapatite NPs suspended in hydrogel	MDA-MB-231 breast cancer cells, MSCs, and MCF-7 breast cancer cells	Breast cancer cells had spherical morphology and migratory characteristics. Co-culture of tumor cells with MSCs increased the formation of spherical clusters. This 3D matrix increased the drug resistance of breast cancer cells compared to 2D culture	[138]
Polyethylene glycol-diacrylate / Hydroxyapatite NPs	MDA-MB-231 / Human fetal osteoblast cell line hFOB	Breast cancer cells interacted with osteoblasts and prevented their proliferation. But osteoblasts stimulated the breast cancer cells growth. Both cell lines increased IL-8 secretion. Breast cancer cells co-cultured with osteoblasts in 3D printed matrix formed multicellular spheroids	[139, 140]
Hydroxyapatite NPs / GelMA / PEGDA	hFob / MDAMB-231 and MCF-7	3D printing provided the possibility of trans-endothelial migration and colony formation for metastatic breast cancer cells. In addition, this model made it possible to evaluate the interaction between cancer cells in a complex vascular microenvironment	[133, 141]
PLA / Hydroxyapatite NPs	MDA-MB-231/ MSCs	The scaffolds were favorable for the growth of metastatic breast cancer cells. Scaffolds with small hexagonal pores had higher cell density than square scaffolds	[142, 143]

Bioinks are derived from various materials, such as natural polymers, synthetic polymers, hydrogels, decellularized ECMs (dECM), tissue spheroids, cell pellets, and microcarriers. dECM derived from patients' tissues plays a vital role in determining cell–cell and cell-ECM interactions, genetic mutations, inducing growth, and differentiation [144, 145]. The hydrogels used for bioink can create 3D architecture with physicochemical and mechanical properties that are adjustable and similar to the native tissue and preserve living cells [129, 133, 146].

The proper design of the bioink to increase cell survival is one of the most significant bioprinting challenges. Other challenges related to the bioprinting method include selecting the appropriate bioink for the tumor tissue, the final model’s dimensions, the time required for modeling, and the need to improve the resolution. The selected bioink must have both the mechanical and physiological properties necessary for the printing process and the printed tumor simultaneously. The development of specific and appropriate bioink in bioprinting can significantly improve drug screening and tumor cells’ interactions in the early stages and progression of various cancers [107, 146].

#### Cancer-on-a-chip systems

Cancer-on-a-chip systems are among the newest tumor models that carefully conserve the patient's tumor characteristics and can be used to select the most effective treatment for the patient. It can determine the success rate of chemotherapy drugs in less than 12 h [147]. Generally, cancer-on-a-chip platforms are 3D and multichannel microfluidic cell culture microdevices that are useful to model the physiology and biology of TME in vitro [116, 148, 149]. The integration of microfluidic technologies, microfabrication methods, and tissue engineering can help make tumor-on-chip systems.

Cancer-on-a-chip systems cause better reconstruction and precise spatio-temporal control of TME parameters. These miniature chips reduce the requirements for sample size and consumable materials during *in-vitro* tests and enable large-scale applications and rapid sample processing [150]. Due to the small size of the specimens, fewer animals are used for further testing, which considerably eliminates ethical concerns. It is possible to accelerate the research process by running several samples on one device.

Combining patient-derived tissues in these models allows for predicting the patient's drug response and determining the most effective drug with minimal toxicity [151]. Surgery, biopsy, aspiration, and the patient's blood sampling are proper methods for obtaining human cells [152]. A biopsy taken from a patient can spread to several tumors or spheroids that can be cultured on a tumor chip. Cancer cells and healthy cells are integrated into tumor chips and used for microclinical trials. Due to the small number of cells required and the high speed of testing on these chips, these systems provide unique tools to facilitate personal anti-cancer drug development [116, 153].

**Fig. 5:**
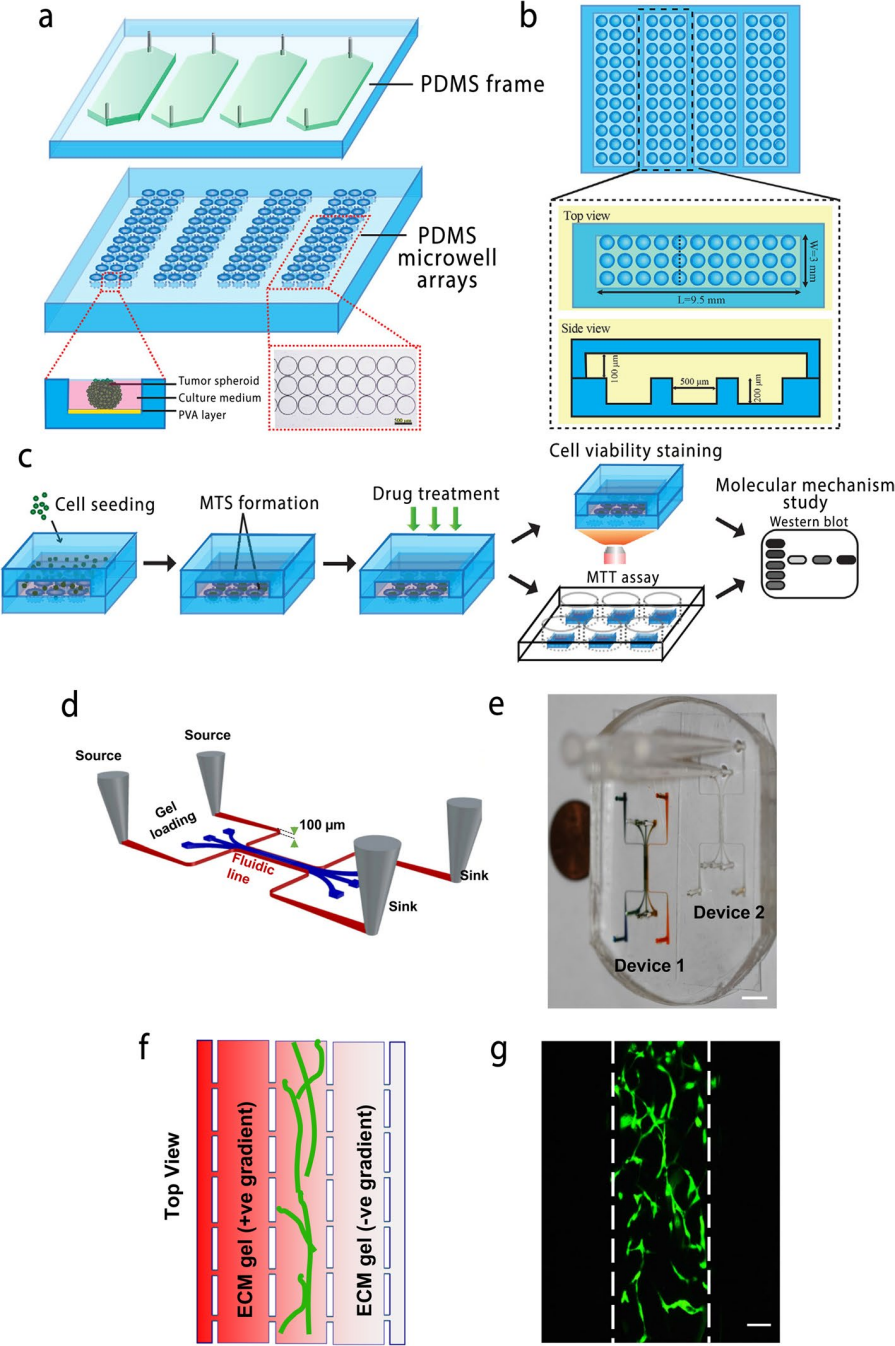
Schematic illustration of the microfluidic device for multicellular tumor spheroids formation and drug screening application; **a** The overall schematic representation of the microfluidic chip. The bottom left displayed the schematic diagram of tumor spheroids formation on a microwell; **b** Top and side views of the microfluidic device; **c** The workflow of multicellular tumor spheroids formation and drug screening-related assay, reprinted with permission from [157]; **d** Schematic diagram of another microfluidic device, which consists of three tissue chambers (blue) and two microfluidic lines (red) connected to sources and sinks. Tiny communication pores (30 μm minimum diameter) allow the microfluidic lines and tissue chambers to communicate via diffusion and convection of interstitial fluid; **e** PDMS platform with two devices. The device on the left has been filled with colored dye to highlight the microfluidic channels. The scale bar indicates 3 mm; **f** The vascular tissue is created in the central chamber while the side chambers are loaded with ECM gels. The pressure and concentration gradients can be created by maintaining differential concentration or pressure in the fluidic lines; **g** Two days after the device is loaded with endothelial cells and fibroblasts, the endothelial cells form a network of the vessel (Green) in the central chamber. Scale bar shows 100 μm, reprinted with permission from [158]

Researchers use transparent glass or polymers, such as polydimethylsiloxane (PDMS), to make these chips [154, 155]. The PDMS material used in fabricating chips is transparent, highly permeable to O_2_ and CO_2_ gases, and made of biocompatible polymers (Fig. 5a-b) [156]. PDMS allows the continuous checking of tissue constructs under a microscope to study cells' behavior and response to treatment (Fig. 5c). Soft lithography, laser cutting, and replica molding methods are conventional approaches to making chips [147, 157]. These chips mimic physiological flow, shear stress, and the delivery of nutrients and drugs like a real tumor by allowing fluid manipulation in small volumes (Fig. 5d-g) [158]. Tumor-derived cells are cultured in small chambers inside these miniature chips to mimic tumor tissue [116, 147].

Applications of cancer-on-a-chip platforms can be summarized as screening anti-cancer drugs and investigating the specific response of the patient's tumor to the drug [159], studying the tumorigenesis processes and the role of the TME in the progression of metastasis [160], conducting research about gene expression [161], creating an accurate mimic of TME elements, noninvasive real-time monitoring of cellular parameters, evaluation of mechanical properties of the TME [162], using it as a model of immunotherapy research [163], and ultimately personalizing cancer chemotherapy [159, 164].

As more than 90% of cancer-related deaths are due to metastasis, metastasis-on-a-chip models are also of particular importance [165]. These models can examine the invasion rate and different stages of the metastatic cascade. This model can be used to evaluate various cancer therapies, such as cell-based therapies, chemotherapy, radiation, anti-angiogenic drugs, antibodies or small molecules, electric field therapy, and targeted nanomedicine [166, 167].

**Fig. 6:**
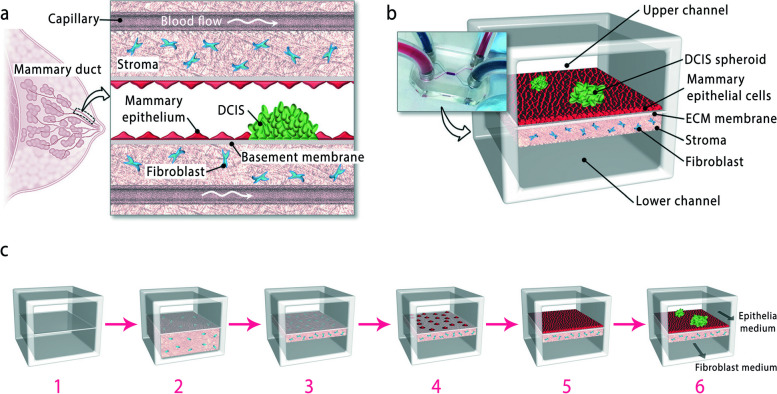
Breast cancer-on-a-chip. **a** Ductal carcinoma in-situ (DCIS) formed in the lumen of the mammary duct, due to the accumulation of neoplastic epithelial cells, in the early stages of breast cancer; **b** Design of the DCIS microarchitecture; **c** Steps of creating DCIS microarchitecture: 1) separation of upper and lower cell culture chambers by collagen membrane. 2) Injection of collagen solution containing human fibroblast cells into the lower chamber 3) Continuous injection of HMF medium from the upper chamber and contraction of the gel due to the tensile force of fibroblasts 4) Create a Matrigel coating on the top surface. 5) Seeding of mammary epithelial cells. 6) Injection and adhesion of DCIS spheroids to epithelial cell, reprinted with permission from [168]

Chen et al*.* [168] studied ductal carcinoma in situ (DCIS) formation via breast cancer modeling utilizing a cancer-on-a-chip system. They developed a biomimetic microengineering strategy to reconstitute the 3D structural organization and microenvironment of breast tumors in human cell-based in vitro models (Fig. 6). Hao et al*.* [167] used a bone-on-chip model to study the bone metastasis of breast cancer. This design simulated the interaction of cancer cells with the bone matrix perfectly. The unique characteristics of breast cancer colonization, previously only confirmed in vivo, were also observed in this model. This model facilitates and replicates laboratory studies of metastasis [167]. Using these chips, researchers can create various cancer-on-chip models and mimic some features in the tumor, such as vasculature, co-culture, shear stress, pressure, mechanical properties, and chemical and oxygen gradients, to better and faster predict drug responses [167].

Microfluidic chips make it possible to mimic a vascular network in a tumor model. Tumor growth and metastasis depend on these networks. All the tumor nutrient and waste exchange processes, migration and metastasis of cancer cells, and delivery of drugs and immune cells to the tumor depend on the tumor’s vascular network [169]. The interaction of vasculature cells with cancer cells has a vital role in regulating TEM and cancer cell phenotype [71, 116, 170].

From the endothelial cell monolayer and circular endothelial cell tube to various cells’ perfusable networks, they can be used to mimic vascularity in cancer microfluidic chips. The monolayer of endothelial cells is efficient when the cylindrical geometry of blood vessels is not necessary. This construction process is simple, has high throughput, and can create shear stress [171]. This monolayer can be formed inside a microfluidic chip by two different methods: sprouting endothelial cells on a porous membrane and in a hydrogel. This strategy is efficacious for studying some drugs that prevent cancer cells from migrating [160, 172].

In more complex cases, two ways can happen: endothelial cells and other cells may grow on a circular scaffold and then be implanted in a microfluidic device, or endothelial cells may grow on the inner surface of a cylindrical hydrogel channel. The vascular of the first manner has mechanical properties similar to those of the natural vascular, but the vascular fabrication method is very tedious. In a second manner, the cancer cells must be cultured in hydrogel before the endothelial cell tube is made [71, 173].

According to the third approach, the sprouting of endothelial cells in the hydrogel causes an irregular vascular network similar to the capillary network. The vessels created in this mode are perfusable. These vascularized tumor chips are efficient for direct analysis of anti-angiogenic and anti-metastatic drugs, modeling the main stages of metastasis, and analyzing physiological barriers that are useful against drug delivery [174].

**Fig. 7:**
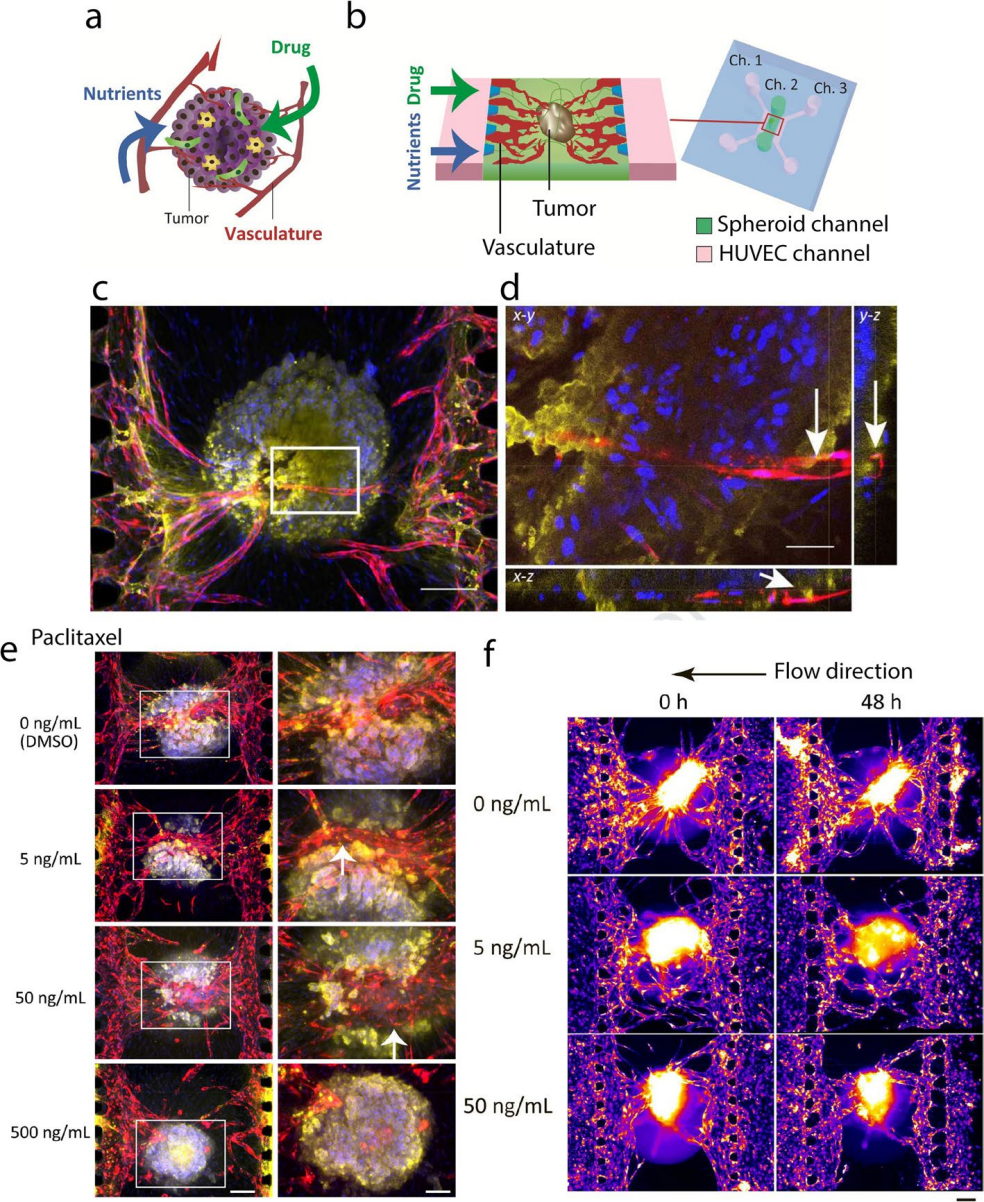
Mimicking chemical and physical microenvironments of tumors in the microfluidic device; **a** In vivo, tumor vasculature serves as the route for nutrients and drugs; **b** Microfluidic platform to recapitulate in vivo tumor microenvironments (TMEs). Immunofluorescence images of the tumor spheroid with the vasculature; **c** Projection image of the tumor spheroid. Scale bar: 200 μm; **d** Orthogonal view from different planes (x–y, x–z, or y–z) of the confocal microscope images corresponding to the white rectangle area in c). White arrows indicate the vascular lumen. Scale bar: 50 μm. Red: RFP-HUVECs, yellow: E-cadherin (Alexa Fluor 633), blue: nuclei (Hoechst 33,342); **e** Drug administration to the tumor spheroids under static 56 condition. 2D projection image (average projection) reconstructed using z-stack images. Red: RFP-HUVECs, yellow: E-cadherin (MCF-7), blue: nuclei. The right column indicates high magnification views of the white rectangle areas in the left column. Scale bars: 200 μm (left column), 100 μm (right column). White arrows indicate E-cadherin-negative areas around the large blood vessels. Drug administration to the tumor spheroids under perfusion condition; **f** Tumor spheroids with the administration of paclitaxel (0, 5, and 50 ng/mL) at 0 h (before administration), and 48 h (after administration for 24 h and incubation with EGM-2 for 24 h). The color indicates the fluorescence of RFP-HUVECs. Scale bar: 200 μm. All the images are reprinted with permission from [175]

The lymphatic system is also involved in the spread of cancer cells. Microfluidic chips mimic this lymphatic system’s role, as well as both transluminal and luminal currents, in increasing cancer cell transmission [170]. Wang et al*.* [173] used sacrificial models of chitosan to make polysaccharide-cellulose-based microtubes. They implanted the resulting porous and elastic vessels in a collagen matrix. Endothelial cells were then cultured on these microtubes’ inner surface, and tumor cells were cultured in a collagen matrix. This model mimics the vascular migration of tumor cells. Nashimoto et al*.* [175] designed a vascularized cancer-on-a-chip platform and investigated perfusion’s effect on tumor growth and drug delivery on this platform (Fig. 7a-b). They found that perfusion in these structures could affect tumor cell culture for more than 24 h and increase their growth. The authors conducted a comprehensive examination of a tumor spheroid by utilizing confocal microscopy to observe the vascularized tumor spheroid. Figure 7c depicts a clear projection image indicating the integration of the tumor spheroid into the vascular network that originated from channels 1 and 3. Figure 7d depicts the observation of a luminal structure in the enlarged orthogonal view of the blood vessel [175].

Also, they pointed out the impact of paclitaxel on tumor spheroids in a static environment. The tumor spheroids with vasculature are depicted in Fig. 7e subsequent to the administration of varying doses of paclitaxel. When the paclitaxel concentration was at 0 ng/mL, the spheroid maintained its spherical morphology and contained a prominent blood vessel at its core, which is analogous to the tumor spheroids observed prior to drug treatment. Conversely, the presence of an E-cadherin-negative region surrounding the sizable blood vessels within the spheroid was detected at concentrations of 5 and 50 ng/mL, as indicated by the white arrows in Fig. 7e. At a concentration of 500 ng/mL, the vascular network exhibited disconnection [175]. They reported that drug administration in the perfusion culture through the vascular network did not decrease the spheroid volume (Fig. 7f). The drug response produced by these systems’ perfusion conditions does not show a dose-dependent effect of the anti-cancer drugs relative to the static conditions [175].

Mixed co-culture microfluidics is useful for creating a heterogeneous cell growth environment in the tumor. These systems mix various cell types and culture them in one chamber. However, in this system, it is difficult to classify different types of monitored cells. In contrast, separate co-culture systems are useful for rapidly recognizing cell types cultured in the device [176]. In these systems, each cell type grows in a separate chamber, and the cells can interact with each other as the media diffuses through the culture medium between these chambers [177].

The cell–cell interactions cannot be transferred through the culture medium, which is not mimicked in this method [71, 178]. Cancer-on-a-chip systems can mimic the tumor’s mechanical properties, such as shear stress, ECM stiffness, and solid tumor elasticity. These properties play a vital role in the development and metastasis of cancer by activating mechanoreceptors. Changes in tumor tissue’s mechanical properties cause changes in the cytoskeleton, changes in the regulation of proteins, and cell behavior changes [179]. Micro- and nano-fabrication methods are efficient in creating these mechanical forces in cancer-on-a-chip systems [180].

Moreover, interstitial fluid pressure (IFP) and solid stress (SS) are two components of extracellular stress that reduce the effectiveness of cancer drugs by creating a barrier to drug delivery. Interstitial flow in the tumor causes a small shear stress, approximately 0.1 Dyn.cm^−2^ [181]. This shear stress has several effects on tumor development. Some of the most important of these include stimulation of oncogenic signaling pathways, upregulation of TGF-β, tightening the ECM by activating fibroblast contraction, and angiogenesis in the opposite direction of interstitial flow [182].

Microfluidic instruments generated a continuous flow of culture medium in the target cells’ direction and simulated this shear stress by using peristaltic pumps, syringe pumps, and the gravitational force due to the difference in flow height at the inlet and outlet of the microfluidic instrument. This shear stress affects the behavior of cells. Modeling this shear stress inside the tumor using microfluidic chips is of great importance [181, 183]. Studies on microfluidic systems have demonstrated the ability of these chips to simulate IFP [71].

The oxygen gradient and chemical gradient in the tumor also affect tumor growth, metastasis, and cancer treatment. An abnormal vascular network and a high density of cancer cells cause a hypoxic core in the tumor. This hypoxia reduces the toxicity of cancer drugs and is involved in malignancy progression through metastasis, so its precise reconstruction in microfluidic chips is essential for drug screening [184, 185].

Diffusion, convection, and electric fields are traditional methods of creating chemical gradients in microfluidic chips [186–188]. Zou et al*.* [189] used two channels with different inlets, one channel containing a target chemical and the other a buffer, to create chemical gradients in microfluidic chips. They seeded lung cancer cells in the chemical gradient formed in the middle of these channels. Their results showed a dynamic response of cells to chemotaxis by the Wnt signaling pathway [189]. Impermeable oxygen materials, gas supply channels near the cell chamber, and perfusing oxygen-scavenging chemicals are used to controlling the oxygen gradient in these chips. Hypoxic incubators are also useful in creating hypoxia [190, 191]. Since PDMS is highly permeable to O_2_ gas, another approach to controlling the oxygen gradient is to replace the PDMS with oxygen-impermeable materials [166, 191].

Acosta et al*.* [191] designed a microfluidic device that can mimic the tumor’s oxygen gradient and create chronic and intermittent hypoxia. They examined the migration of cancer cells on this platform and showed that hypoxia causes a more aggressive phenotype. To design this model, they used O_2_ gas emissions between the two gas supply channels. Aung et al*.* [192] developed a tumor-on-a-chip model using micro-patterning integration with microfluidics. They inserted a combination of endothelial cells and cancer spheroids into the gelatin methacrylate (GelMA) hydrogel inside the chip. They used the different motility of endothelial and cancer cells in response to a controlled morphogen gradient across the network to control the organization within microfluidic chips. They found that the migration of cancer cells depended on the location of the chemical source.

These systems are currently mostly used in research and have not yet seriously entered the clinical and industrial phases. The development of these chips in the clinical space has faced some limitations. Also, there is still no easy access to patient-derived tissues. Making these chips requires high levels of skill and experience. The integration of other techniques, such as organoid and 3D printing, into these chips is useful in their development. 3D printing technology facilitates the easier production of complex and functional chips.

### In vivo* models*

Bone metastasis is a complex and multistep process involving a variety of signaling pathways. During metastasis, tumor cells undergo genetic and phenotypic changes and interact with other cells in the microenvironment [193, 194]. To create bone metastasis, tumor cells grow in the primary site, and by secreting various factors, they help prepare the bone for the entry of tumor cells. Next, the cells undergo an epithelial-to-mesenchymal transition. They spread in the blood circulation, and when they reach the bone, they are removed from the blood circulation and implanted in the metastatic niche of the bone. Generally, these cells undergo a period of sleep, and then, by interacting with the bone microenvironment, they help to reduce the anti-tumor immune response and create osteolytic lesions. Therefore, during metastasis, all the organisms of the body are involved [195, 196].

It is essential to provide suitable animal models to determine the pathogenesis of bone metastasis, identify suppressors and genes involved in metastasis, conduct chemotherapy studies, and provide new treatments for bone metastasis [197]. Animal models are the gold standard for studies related to metastasis and cancer treatment, but unlike humans, in whom spontaneous metastasis of breast tumors to bone is common, metastasis to bone is less common in animal models [24, 198]. In addition, ethical concerns, the high cost, and the different metabolic characteristics of animals have limited their use. To date, no model has reproduced all the genetic and phenotypic changes of breast cancer bone metastasis [199]. The type of tumor, ease of use, time, cost, extent of the immune system, and degree of similarity to the process of bone metastasis in humans are key parameters in choosing an animal model [200].

Animal models are classified based on various parameters, such as species, immune status of the host, site of implantation, and mode of tumor formation. Spontaneous tumorigenesis, carcinogenic agents, genetic manipulation, and transplantation of breast cancer cell lines have been used to produce animal models of breast cancer bone metastasis in rodents (with complete immunity and immunodeficiency) and non-rodent animals (such as Zebrafish and Drosophila melanogaster) [201]. Transplantation of tumor cells creates allograft (syngeneic) and xenograft models [202–204]. Xenograft tumor models, in turn, are divided into two categories: cell-derived xenografts (CDX) and patient-derived xenografts (PDX) [198, 205].

Bone metastases in breast cancer models are generally induced by injecting tumor cells into the site of metastasis (bone tissue) or other sites such as the heart [198, 206], blood circulation, adipose tissue [207], and caudal artery and veins [208, 209]. Each of these sites has a different bone metastasis efficiency. Generally, tail vein injection causes lung metastases, and intracardiac injection causes bone and brain metastases [210]. Various positive estrogen-receptor (ER +) cells (such as MCF-7) and negative estrogen-receptor (ER-) cells (such as MDA-MB-231 triple cells) have been used to model bone metastasis [211]. Generally, ER + cells use the intracardiac injection route for modeling. The creation of osteolytic and osteoblastic lesions by these cells is challenging and requires a lot of time. In contrast, ER-cells metastasize to the bone within 2–4 weeks, creating osteolytic lesions [212].

To model the growth of tumor cells in the primary site and spontaneous metastasis to bone, tumor cells can be injected into the mammary fat layers of mice, which leads to bone metastasis in 40–60% of cases [207]. The injection of breast cancer cells into the bloodstream can be used to investigate homing, dormancy, colonization, tumor growth, and interactions related to the bone microenvironment [213, 214]. Injection of human MDA-MB-231 cells or mouse E0771 cells into the left ventricle of the heart in mouse strains leads to homing in the long bones of the spine. To increase the homing of bone cells and reduce the need for intracardiac injection, MDA MB-231 subtypes obtained from repeated in vivo passages from mouse bone can also be used. Tail injection into these subcategories has led to bone metastasis in 95% of cases. MDA MB-231 subtypes gave rise to tumors in 90% of mice after injection into the tail artery, whereas MDA-IV cells gave rise to tumors in 80–90% of mice after injection into the lateral tail vein [60, 215]. These metastases had constant size and position and minimal metastasis to vital organs. Therefore, the mice maintained their health for a longer period. This method led to a reduction in the total number of animals used and related costs. It should be noted that the age of the animal (5 to 8 weeks) is also essential for achieving bone metastasis following intracardiac, intravenous, or intraarterial injection.

The intra-tibial injection is used to model the final stages of breast cancer bone metastasis and the direct interactions between tumor cells and the bone microenvironment. Injection of 10,000 4T1 cells into the tibia results in osteolytic lesions, and increased cell concentrations lead to metastasis to the femur, lungs, and forelimbs [196]. Direct injection into bone tissue accelerates modeling and ignores metastatic processes. On the other hand, routes such as the heart and blood circulation lead to the tumor cells in the bone, preparing the microenvironment for tumor development and progression. The engraftment of trabecular bone fragments taken from the patient's femoral head in mice can be used to investigate metastasis in the human bone environment. Injecting MDA-MB-231 or SUM-1315 subtypes in these mice leads to metastasis in human bone implants after four weeks of implantation [216, 217]. This method is valuable for modeling the interactions between the bone microenvironment and tumor cells in immunocompromised mice after injection. This method has the advantage of a high rate of tumor uptake in bone and is beneficial for studying genetic manipulation of the host/tumor cell environment.

However, each of these paths has limitations. Intracardiac injection of cancer cells is difficult and does not lead to specific bone metastases. On the other hand, intravenous injection generally leads to the production of lung tumors that rarely metastasize to the bones (usually metastasizing to the liver, spleen, or brain). In addition, large lung tumors mask weaker signals in other parts of the body. Injection into fat results in a low rate of metastases to bones and increases the number of animals needed. Intraosseous injections are also well controlled in terms of cell growth, but due to the need to drill bone, they cause local inflammation that does not mimic the metastatic process of cancerous bone from the circulatory system [208, 218]. Therefore, choosing your cell line and inoculation route should be based on dose–response studies before starting large animal experiments.

Based on the immune status of the host, animal models can be classified into two categories: immunocompetent and immunodeficient. Immunocompetent animals provide a complete immune system, and modeling breast cancer bone metastasis in them can evaluate the immune system's interaction with different stages of the metastatic process and anticancer agents [219]. Furthermore, these models can investigate lytic disease and mixed lesions, but these syngeneic models do not allow the use of human breast cancer cells or PDX [220]. BALB/c, C57BL/6, and FVB mice are used for mouse cell transplantation, carcinogen induction, and genetic modification [221]. Breast cancer, the bone microenvironment, and activated immune cells differ in humans and mouse. Human breast cancer metastasizes to the bone most of the time (80%), but mouse breast cancer mainly metastasizes to the lung and rarely to the bone [222]. These low rates of spontaneous bone metastasis have several reasons. Mouse and humans have biological differences. Mouse cells have more metabolic activity and a longer telomerase than human cells, which affects oncogenesis and phenotypic differences. Mouse tumors have the origin of mesenchymal tissue, and human tumors have the origin of epithelial cells. Furthermore, murine mammary tumors are not hormone-dependent, whereas most human breast tumors (especially those that metastasize to bone) are hormone-dependent and require higher concentrations of estrogen to support their growth [222, 223]. Therefore, the data from these models should be interpreted based on these differences.

To address this issue, researchers have generated bone trophic subtypes of mouse breast cancer cells through repeated passage in vivo from the bone. Injection of some of these cell lines into rodent strains resulted in successful metastasis to mouse bones (e.g., 4T1 (20%), E0771 (60–80%), and KEP (50%)) [59, 224, 225]. The injection of these cell lines into the left ventricle of the heart or bone has led to significant metastases in the bone. However, due to the rapid metastasis of syngeneic lines to the lung and its rapid growth, the primary tumors should be surgically removed to have the necessary time for metastatic detection in the bone because metastatic bone deposits are small and undetectable [24, 226].

Immunodeficient mouse models are divided based on their immunological profiles. Immunodeficient mouse models used in breast cancer bone metastasis include BALB/c nude, MF1 nude, NOD SCID, and NSG, each unique in terms of primary tumorigenesis and metastatic potential [196]. Using immunodeficient rodents helps facilitate the growth of human breast cancer cells in the host. These models help to study human cells in a host environment by eliminating confounding effects related to the animal's immune response. These mice lack a thymus and cannot produce mature T lymphocytes. Therefore, the possibility of rejecting the transplant is low for them [227]. These mice are used to study tumor cell colonization in bone, stages of metastasis, metastatic dormancy, and tumor growth [213, 228]. ER-cells with the ability to rapidly create osteolytic lesions are the first choice for the formation of bone metastasis in these models [213, 228]. However, since most types of human breast cancer that metastasize to bone are ER-positive, various studies have also used these cells. ER-positive cell metastasis to bone causes the formation of mixed lesions, and estradiol supplementation is needed to stimulate their growth in a non-human environment. These supplements change the bone microenvironment and make data interpretation difficult. ER-positive cells require times longer than 6 months (long-term dormancy in bone) to metastasize to bone in the absence of estradiol in the bone of immunodeficient mice. This model of ER-positive breast cancer contributes to our knowledge of dormancy and metastatic growth [229]. PDX xenografts also grow only in severely immunodeficient (NOD-SCID) mice. In NOD-SCID mice, the number of T and B lymphocytes, granulocytes, natural killer cells, and macrophages and their functions are reduced [198, 230]. But considering the role of the immune response in tumorigenesis and the activity of anticancer agents, these models do not mimic the body condition in the metastatic process [231, 232].

Non-rodent animals are also another option for modeling bone metastasis. Zebrafish have been developed as a non-rodent in vivo model to study human tumor growth, metastasis initiation, angiogenesis, and interaction with the microenvironment. Due to the rapid external growth of transparent zebrafish embryos and the ease of their genetic manipulation, zebrafish have become an excellent in vivo model for investigating single-cell interactions and the signaling mechanisms involved [233, 234]. Fluorescently labelled cancer cells can be transplanted into zebrafish embryos without worrying about transplant rejection. This in vivo model allows studying the behavior of various breast tumor cell lines with different bone metastatic potentials, PDX, and anti-metastatic drug treatments [234, 235]. Melanogaster is another non-rodent model for breast metastasis studies that provides a suitable platform for high-throughput genetic screening [236]. Among the above-mentioned models, PDX allows a more accurate summation of the phenotypic and genetic characteristics of tumors. Therefore, it will be discussed in detail in the next section.

#### Patient-derived xenograft (PDX)

The history of disease modeling for drug screening goes back to the 1950s, when researchers used different cell lines to induce disease-like conditions in animals [237]. CDX served as the gold standard model for decades; however, it has gradually become known that these models’ genetics and architecture vary significantly across different cell lines, various in vitro expansions, and various laboratory environmental conditions. Notably, the expansion of cells in vitro for months or even years causes a significant change in the cells’ molecular characteristics, and they do not resemble their parental tumors following the injection into animal bodies [238, 239].

**Fig. 8:**
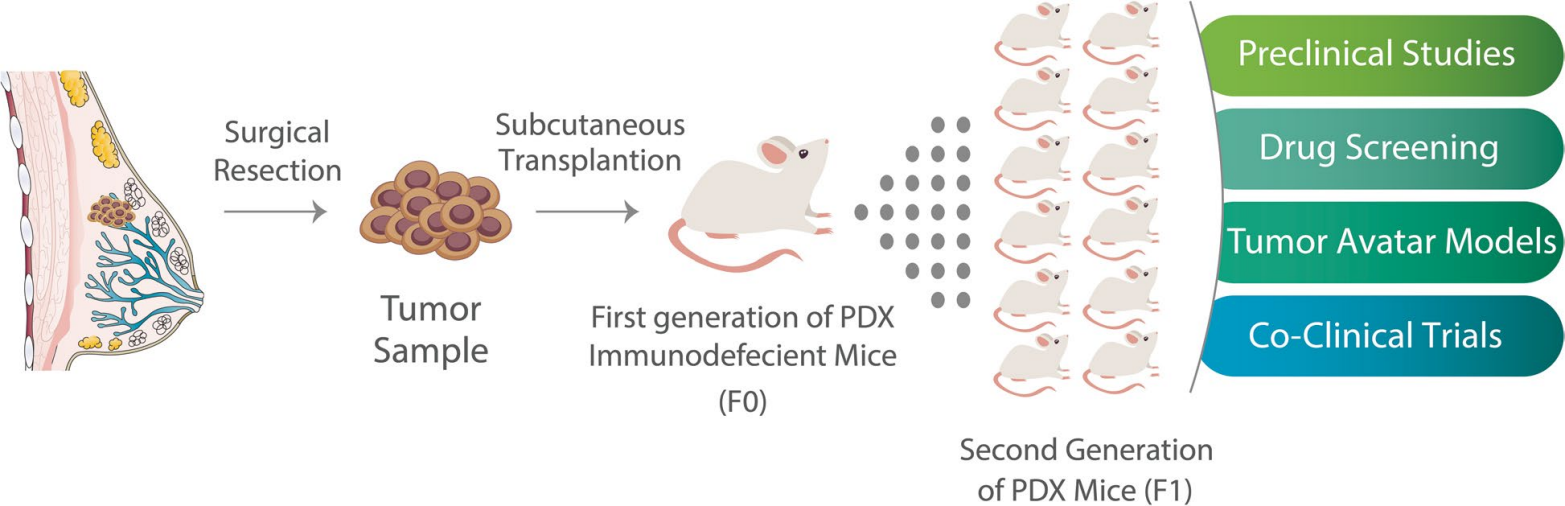
Patient-derived xenografts (PDX) and their applications

Although the CDX has remained a valuable animal model since 1969, the PDX was established as a useful tool for mimicking human tumors [83]. Transplanting a few human tumors into small subcutaneous pockets in immunodeficient mice allows them to grow (Fig. 8) [240]. Transplanting this minced tumor of the first generation of mice into several recipient mice gives a conservative model that maintains the original human parental tumor regarding genetic, epigenetic, pathological, and molecular features (Table 3) [241, 242].

**Table 3:** Recent patient-derived xenografts by tumor type, immunodeficient mice, and site of implantation

Animal	Site	Ref
Zebrafish	duct of Cuvier	[234, 243]
NOD-SCID	Orthotopic	[244]
Nude	Orthotopic	[245]
NOD-SCID	Subcutaneous	[246]
NSG	Subcutaneous	[247]
NSG	Orthotopic	[248]

Molecular and cytogenetic analysis of PDX showed significant resemblance to their parental tumors [243, 249]. The PDX response to anti-cancer treatments was highly comparable with clinical settings, which provides us with a unique opportunity to develop personalized medical treatments [250, 251]. Compared to their new comparator (organoids), in addition to their 3D propagation, the ability to evaluate them in vivo provides the conditions for researchers to study systemic changes in disease and their designated treatment effects. Also, numerous cells result from subcutaneous growth in the recipient mice's suitable environment, providing a reliable and replicable model on small and large scales [170, 252, 253].

Besides, one of the primary differences between CDX and PDX was the presence of tumor stroma, which supports the integrity and flexibility of the tumor and mediates perfusion, cell signaling, and cellular kinetics [254]. The application of PDX models is moving beyond preclinical studies. PDX models highly resemble human tumors regarding identification, monitoring, and treatment biomarkers [255]. They were incorporated into the clinical studies as avatar models of human tumors to assess the sensitivity and efficacy of anti-cancer therapies in clinical studies called co-clinical trials [256–258].

**Fig. 9:**
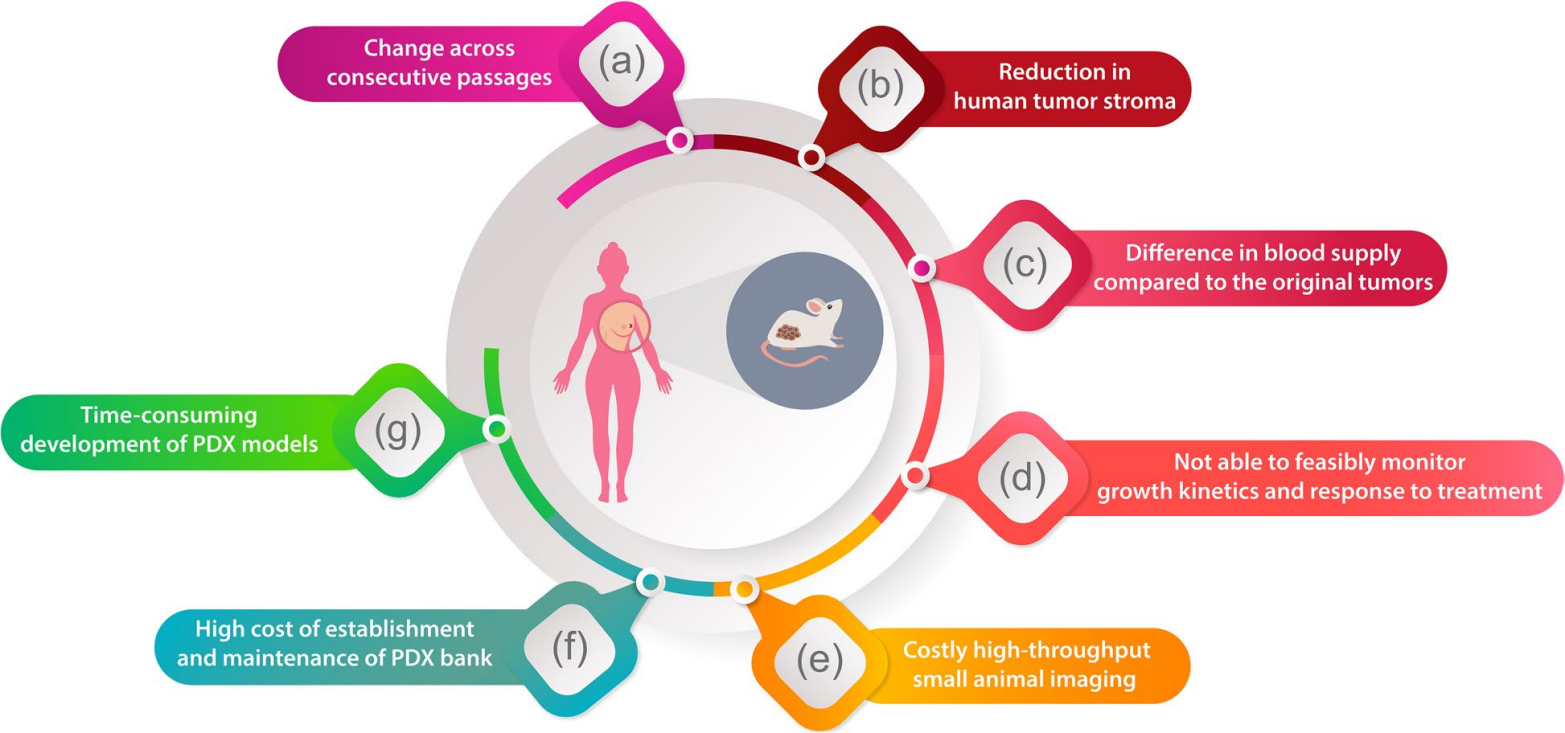
Limitations and obstacles of patient-derived xenografts tumor models

Currently, an increasing number of preclinical studies are using PDX models [240]. Along with the numerous advantages of PDX models, there are still a few obstacles that need to be overcome (Fig. 9). The inability to evaluate the immune system is one of them. As mentioned before, highly immunodeficient mice, such as NSG mice, are used for developing PDX models. Lack of immune system compartments, such as natural killer cells, B, and T lymphocytes in NSG mice, limits the ability to assess the effect of immune modulator drugs and immunotherapies focused on the recipient immune system, like vaccines [257].

The replacement of human tumor stroma with murine stroma is another major issue. After approximately 3–5 passages of PDX models getting ready for drug screening, the transplanted tumor stroma would be replaced wholly with murine connective tissue [259]. Due to the paracrine effects of the stroma and species-specific cytokines, this stromal replacement leads to heterogeneity in the tumor and can confound the findings [169]. Also, the tumor intake rate, which is defined as the chance of engrafting the implanted tumor pieces, was still low in different settings [260]. The use of a support matrix, including growth factors, can increase the engraftment success rate, alter the ECM regulatory interactions, and therefore artificially affect the tumor kinetics [169, 261].

Another significant parameter is time-consuming models, which need about 4–8 weeks to develop PDX models for personalized medical decision-making. This issue would limit its usage. Moreover, the site of transplantation is considered a primary factor. The surrounding environment has a significant effect on tumor behavior. Hence, implanting the tumor pieces in their original anatomic site (orthotopic) versus implantation in subcutaneous or sub-renal areas leads to different tumor behaviors, therefore altering the treatment response. Also, the implantation site plays a pivotal role in engrafting the tumors with a smaller size than the original tumors [262].

## Challenges and future perspective

Bone metastasis (the most common site of breast cancer metastasis) affects the survival rate and quality of patients' lives. Therefore, providing models to understand the mechanism and mode of breast cancer's bone metastasis, drug screening, evaluation of drug release carriers, and development of new treatments to prevent the destructive effects of bone metastasis are among the main clinical challenges. So far, various in vitro and in vivo models have been developed to study breast cancer's bone metastasis.

In vitro models mimic the tumor microenvironment, investigate cell-microenvironment interactions, and evaluate tumor therapeutic responses [216]. Various 2D and 3D in vitro models have been proposed. Monolayer (2D) cultures are generally developed to study the migration and invasion of cancer cells and drug evaluations through a porous membrane [263]. In these models, the patient's cells (resulting from biopsies) can be used [264]. Animal models have also tried to model metastasis events and help in their treatment by using genetic manipulation or injection of tissues, cancer cells, and carcinogens in various animals with complete immunity and immunodeficiency. Current animal model systems generally do not represent the dormant phase of cancer cells [202, 226].

Of course, with methods such as intracardiac injection of human tumor cells in adult animals, it is possible to mimic the delay time of breast cancer's bone metastasis. However, this remains a challenge due to the need to remove the primary tumor to allow enough time for bone metastasis to develop, as well as the multiple possible sites for metastasis and their different growth kinetics [213].

Generally, immunodeficient mice are not able to mimic human immunology and stromal interactions, and expensive human mice and PDX should be used. In addition, new animal models are needed to study the bone metastasis of male breast cancers and the effect of various factors, such as menopause, on the therapeutic responses of bone metastasis. Unfortunately, these studies require more animals and more money, which is not economically and ethically justified [265].

As the next generation of bone metastasis models, 3D models (including spheroids, organoids, and scaffolds) have been proposed to overcome the problems related to 2D models (static condition) and animal models (high cost, ethical problems, and different physiology) and provide an accurate, reliable, and efficient model for evaluating bone metastasis in breast cancer. These developing models seek to provide better indicators to investigate the mechanism of metastasis and the effectiveness of drugs in vivo. Of course, current 3D models also face limitations.

Current spheroid models do not have a uniform size and, consequently, an un-uniform distribution of nutrients, leading to uneven cell growth in the spheroids [266]. The materials used in the scaffold fabrication are also effective in increasing the absorption rate and screening nature of anti-cancer drugs. In general, the current models are relatively simple and usually resemble the tumor in terms of morphology and differ from it in phenotype and heterogeneity [267]. These models are generally established with long-term culture-adapted cell lines that may not adequately match the pathology of bone metastasis [73]. Furthermore, the different culture conditions required for the various cells, the autofluorescence of the scaffold materials when imaging the cells, and the genetic and epigenetic changes of the cells over time are other problems with these systems [268]. Therefore, the standardization and automation of 3D models are requirements for their future applications. Creating more complex culture systems that can recreate organ functions and dynamic microenvironments in the future can be clinically useful for biological processes related to primary and metastatic tumors and the evaluation of their therapeutic responses to various types of drug carriers and new drugs.

Therefore, the next generation of in vitro tumor models will integrate new technologies into existing models. In fact, in future studies, systematic studies using artificial intelligence can be used to predict cell behavior based on the chemical composition, geometry, and mechanical characteristics of substrate materials [269, 270]. These algorithms provide the possibility of a quick and accurate initial description of 3D models to develop suitable substrates for the growth of cancer cells. In fact, by using these algorithms and 3D models, it is possible to predict the progress and metastasis of cancer [271].

## Conclusions

The occurrence of bone metastasis poses a significant obstacle for individuals with breast cancer, and a range of in vitro and in vivo models have been established to investigate this phenomenon. In vitro models are utilized to replicate the complex tumor microenvironment, explore the interactions between cells and their surrounding microenvironment, and assess the efficacy of therapeutic interventions for tumors. The replication of the latency period of bone metastasis through animal models has posed a challenge, primarily due to the requirement of primary tumor removal and the existence of numerous potential metastatic sites. Novel bone metastasis models, such as three-dimensional (3D) models, have been suggested as a potential solution to address the limitations associated with two-dimensional (2D) models and animal models. Notwithstanding, extant 3D models are constrained by factors such as irregular cellular proliferation, autofluorescence, and alterations in genetic and epigenetic expression. Standardization and automation of 3D models are imperative for enhancing their future applications. The application of artificial intelligence has the potential to forecast cellular activity by analyzing the chemical composition, geometry, and mechanical properties of substrate materials. The utilization of these algorithms has the potential to forecast the advancement and dissemination of cancer.

## Data Availability

All the data that support the findings of this study are available in this manuscript.

## Abbreviations

CTGF: Connective tissue growth factor
COX: Cyclooxygenase
CSC: Cancer stem cell
CAFs: Cancer associated fibroblasts
CDX: Cell-derived xenografts
DC: Dendritic cell
dECM: Decellularized ECMs
DCIS: Ductal carcinoma in-situ
EMT: Epithelial-mesenchymal transition
ECM: Extracellular matrix
FGF: Fibroblast growth factors
GAG: Glycosaminoglycan
GelMA: Gelatin methacrylate
HTS: High-throughput screening
HCI: High-content imaging
IGF: Insulin-like growth factors
IFP: Interstitial fluid pressure
LIF: Leukemia inhibitory factor
MDSC: Myeloid-derived-suppressor cells
MMP: Matrix metalloproteinases
MSC: Mesenchymal stem cells
MAP: Mitogen activated protein
MDR: Multidrug resistance
NK: Natural killer
PTH-Rp: Parathyroid hormone-related peptide
PGE2: Prostaglandin E2
PEG: Polyethylene glycol
PDMS: Polydimethylsiloxane
PDX: Patient derived xenograft
RANKL: Receptor activator of nuclear factor-κB ligand
SRE: Skeletal related events
SS: Solid stress
TGF-β: Transforming growth factor-β
TNF-a: Tumor necrosis factor-a
TME: Tumor microenvironment
TAM: Tumor associated macrophages
VEGF: Vascular endothelial growth factor
